# Artificial intelligence-driven computer aided diagnosis system provides similar diagnosis value compared with doctors’ evaluation in lung cancer screening

**DOI:** 10.1186/s12880-024-01288-3

**Published:** 2024-06-11

**Authors:** Shan Gao, Zexuan Xu, Wanli Kang, Xinna Lv, Naihui Chu, Shaofa Xu, Dailun Hou

**Affiliations:** 1 Beijing Tuberculosis and Thoracic Tumor Research Institute, Beijing, China; 2 Beijing Chest Hospital, Capital Medical University, Beijing, China

**Keywords:** Artificial Intelligence, Computed Tomography, Pulmonary nodule, Lung cancer, Diagnosis

## Abstract

**Objective:**

To evaluate the consistency between doctors and artificial intelligence (AI) software in analysing and diagnosing pulmonary nodules, and assess whether the characteristics of pulmonary nodules derived from the two methods are consistent for the interpretation of carcinomatous nodules.

**Materials and Methods:**

This retrospective study analysed participants aged 40–74 in the local area from 2011 to 2013. Pulmonary nodules were examined radiologically using a low-dose chest CT scan, evaluated by an expert panel of doctors in radiology, oncology, and thoracic departments, as well as a computer-aided diagnostic(CAD) system based on the three-dimensional(3D) convolutional neural network (CNN) with DenseNet architecture(InferRead CT Lung, IRCL). Consistency tests were employed to assess the uniformity of the radiological characteristics of the pulmonary nodules. The receiver operating characteristic (ROC) curve was used to evaluate the diagnostic accuracy. Logistic regression analysis is utilized to determine whether the two methods yield the same predictive factors for cancerous nodules.

**Results:**

A total of 570 subjects were included in this retrospective study. The AI software demonstrated high consistency with the panel's evaluation in determining the position and diameter of the pulmonary nodules (kappa = 0.883, concordance correlation coefficient (CCC) = 0.809, *p* = 0.000). The comparison of the solid nodules' attenuation characteristics also showed acceptable consistency (kappa = 0.503). In patients diagnosed with lung cancer, the area under the curve (AUC) for the panel and AI were 0.873 (95%CI: 0.829–0.909) and 0.921 (95%CI: 0.884–0.949), respectively. However, there was no significant difference (*p* = 0.0950). The maximum diameter, solid nodules, subsolid nodules were the crucial factors for interpreting carcinomatous nodules in the analysis of expert panel and IRCL pulmonary nodule characteristics.

**Conclusion:**

AI software can assist doctors in diagnosing nodules and is consistent with doctors' evaluations and diagnosis of pulmonary nodules.

## Introduction

Lung cancer is not only the leading type of cancer and cause of death worldwide, but it is also the most prevalent form of cancer in China [1, 2]. Computed tomography (CT) is the most commonly used non-invasive examination for lung cancer screening. Low-dose CT screening of pulmonary nodules can help detect lung cancer at the early stage. Several studies have shown that low-dose CT screening of the chest is beneficial in reducing lung cancer mortality [3, 4].

The doctors assessed the screening nodules' features of size, location, attenuation characteristics, and morphological signs. They combine these features with clinical information to determine whether the nodules are benign or malignant. Although radiologists use standard terminology, which was recognized by organizations such as the Fleischner Society and the Radiological Society of North America, to describe pulmonary nodule types and morphological features, the assessment of nodules is still subjective and empirical, and they cannot be interpreted objectively in a quantitative manner [5]. The workload of radiologists who interpret large number of chest CT scans each day can result in overlooked and incorrectly diagnosed nodules [6].

The recent surge in artificial intelligence (AI) has resulted in rapid advancements in medical imaging. AI algorithms and functions now enable a more objective representation of lesion signs. Numerous research findings have emerged in the field of AI in medicine, particularly in pulmonary nodules. Several studies have shown that AI-assisted CT diagnostic techniques have demonstrated strong diagnostic performance in distinguishing between benign and malignant pulmonary nodules using various classification algorithms. The sensitivity ranges from approximately 78.90% to 96.00%, and the accuracy from 84.6% to 95.41% [6–8]. However, some algorithms are not used for large-scale lung cancer screening [9].

There is an AI-driven commercial computer-aided diagnostic(CAD) product (InferRead CT Lung, IRCL) based on a three-dimensional (3D) convolutional neural network (CNN) with DenseNet architecture. This product can detect pulmonary nodules and characterize the various properties of the nodules. This study aims to: 1) analyse the consistency of CT images of subjects with ≥ 5 mm pulmonary nodules interpreted by doctors and AI software in lung cancer screening for location, attenuation characteristics, the maximum diameter, and morphologic characteristics; 2) further compare and analyze the superiority of the two modalities, doctors and AI, in identifying benign and malignant pulmonary nodules, using pathological findings as the gold standard; 3) identify factors for predicting cancerous nodules, determine if they are consistent for nodule characteristics between doctors and the AI software, and assess the potential of an AI-driven computer-aided diagnostic system for lung cancer screening.

## Materials and methods

### Patients enrollment

Using the population data collected from 2011 to 2013 as part of the Beijing Science and Technology Project-Baseline Survey of Lung Cancer in Beijing and Study on Early Prevention and Treatment Strategies (Project No. Z151100002115049), an expert panel consisting of radiologists, oncologists, and thoracic surgeons simultaneously evaluated and analysed pulmonary nodules ≥ 5 mm in the study population, leveraging their clinical experience. Due to the extremely low incidence of lung cancer in individuals under 40 years of age, the subjects aged 40–74 years would process pulmonary nodule screening.

Information on whether the study participants had a family history of smoking, whether they smoked (≥ 20 packs/year), whether they had passive smoking, whether they had chronic bronchitis, and a history of occupational exposure (including by-products of aluminum products, arsenic, asbestos, chromium compounds, coke ovens, mustard gas, nickel-containing impurities, vinyl chloride) was recorded. The individuals who exhibited suspected symptoms or risk factors for lung cancer had their blood samples collected and underwent low-dose chest CT examinations and tumor marker examinations, which included carcinoembryonic antigen (CEA), neuron-specific enolase (NSE), and cytokeratin 19 fragment antigen 21–1 (Cyfra21-1).

Subjects underwent a low-dose chest CT scan, then followed by panel evaluation and IRCL analysis. Characteristics of the pulmonary nodules were recorded, including their location (upper right lobe, middle right lobe, lower right lobe, upper left lobe, lower left lobe), size measurement (the maximum diameter), attenuation characteristics (solid, subsolid, nonsolid), and morphological characteristics (spiculation sign, lobulation sign, pleural indentation sign). We confirm that all methods performed adhere to the relevant guidelines and regulations.

### Inclusion and exclusion criteria

The inclusion criteria were: (1) local household registration and permanent residence in Beijing (residing for more than ten years); (2) the subjects aged 40–74 years; (3) the presence of pulmonary nodules with a diameter of ≥ 5 mm on low-dose chest CT screening.

The exclusion criteria were: (1) subjects with severe mental illness or those who are too emotionally disturbed to participate in the survey or unable to answer questions clearly due to illness; (2) subjects with missing contact information in the Beijing Tumor Registry database and cannot be reached; (3) patients with a history of confirmed lung cancer; (4) patients without pulmonary nodules; (5) patients with pulmonary nodules smaller than 5 mm in diameter; (6) history of other neoplasms; (7) points to unspecified pulmonary nodules when analyzed by expert panel and IRCL; (8) patients with pulmonary nodules measuring ≥ 30 mm in diameter as measured by the panel and IRCL.

### CT scanning parameters

The Bright Speed Elite (GE Healthcare), Light Speed VCT, Optima CT 680 (GE Healthcare), SOMATOM Definition AS (Siemens Healthineers), and Brilliance (Philips Healthcare) would be utilized for low-dose chest imaging. The patients were positioned flat, and the scan area covered the lung tip to the base of the lung. The following parameters were: (a) tube voltage at 120 kV; (b) current adjusted using an automatic technique (range 20-50mAs for Bright Speed Elite, Light Speed VCT, Optima CT 680, Philips, and 30mAs for SOMATOM Definition AS, Siemens); (c) layer thickness/spacing of 5 mm; (d) pitch of 1.375 for Bright Speed Elite, Light Speed VCT, Optima CT 680, Philips, and 1.3 for SOMATOM Definition AS, Siemens; (e) lung window reconstruction with a slice thickness/spacing of 1.25 mm/1.25 mm; (f) lung window reconstruction with a slice thickness/spacing of 1.25 mm/1.25 mm and a window width/position of (1500,-500) HU; mediastinal window reconstruction with a layer thickness/spacing of 1.25 mm/1.25 mm and a window width/position of (400,40) HU.

### Detection by a computer-aided diagnostic system

The IRCL developed by Beijing Infervision Technology Co., Ltd. (Beijing, China). It is based on a 3D CNN with DenseNet architecture as a backbone (without clinical data). This product uses the Faster R-CNN model to detect nodules. The AI model utilized a region-based CNN for object detection, comprising two modules. The first module was the Regional Proposal Network(RPN) designed to generate object proposals by a convolutional network. The second module was the Fast R-CNN detector, which aimed to enhance the proposals by the first module generated.

### Diagnosis of lung cancer

Lung cancer is diagnosed through histopathological examination of the resected specimen. Before surgical resection, nodules are marked using microcoil localization techniques under CT guidance. The excised tumor is classified according to the lung tumor classification of the National Health Commission of the People’s Republic of China (WS 323–2010).

### Data analysis

SPSS 26.0 and MedCalc 16.8.4 were utilized for statistical analyses. Comparisons between continuous variables were made using the Mann–Whitney U test. Comparisons between categorical variables were made using the χ^2^ test, and unordered categorical variables were evaluated using Cohen's Kappa concordance test. Concordancy for continuous variables was assessed using the concordance correlation coefficient (CCC) and Bland–Altman analysis. The diagnostic effectiveness of doctors' and IRCL was evaluated by receiver operating characteristic curves (ROC). The area under the curve (AUC) and 95% confidence intervals(CI) illustrated the comparative results of these two methods. Factors for interpreting carcinomatous nodules based on pulmonary nodule characteristics were analysed using univariate and multivariable logistic regression. The maximum diameter was converted to categorical variables using boundaries of 8 mm, the Hosmer–Lemeshow test was used to assess model fit. The p-value < 0.05 was considered to indicate a statistically significant difference.

## Results

### Patients recruitment

Participants were enrolled based on the subject inclusion and exclusion criteria from 2011 to 2013. A total of 744 cases with pulmonary nodules of ≥ 5 mm in diameter were screened using low-dose chest CT. Among them, 166 cases were excluded due to multiple instances of pointing to unknown pulmonary nodules, and 8 cases were identified by an expert panel and IRCL as lesions with a diameter of ≥ 30 mm. Ultimately, 570 subjects were enrolled. The largest number of subjects were aged 60–69 years (45.26%, 258/570). A total of 153 participants smoked, with a higher proportion of men (≥ 20 packs/year) than women (χ^2^ = 167.192, *p* = 0.000). There was no statistical difference between males and females in the detection of tumor markers, with p-values of 0.206, 0.537, and 0.115, respectively. Out of the 570 subjects included in the study, 41 were confirmed to have lung cancer. Among them, 22 (53.66%, 22/41) participants had adenocarcinoma, 14 (34.15%, 14/41) participants had squamous carcinoma, and 5 (12.20%, 5/41) participants had other types of lung cancer. There were more women than men with lung cancer (χ^2^ = 4.321, *p* = 0.000), and the same trend was observed in adenocarcinoma cases (χ^2^ = 14.421, *p* = 0.000). (Table 1).

**Table 1:** Demographic and clinical characteristics of the study participants

Characteristics	male *n* = 278(%)	female *n* = 292(%)	*P* value
age(year)			
40–49	21(7.55%)	35(11.99%)	0.076
50–59	94(33.81%)	102(34.93%)	0.736
60–69	131(47.12%)	127(43.49%)	0.384
70–74	32(11.51%)	28(9.59%)	0.455
smoking (≥ 20 packs/year)	143(51.44%)	10(3.42%)	**0.000**
passive smoking	148(53.24%)	148(50.68%)	0.542
occupation	6(2.16%)	10(3.42%)	0.360
lung cancer family history	32(11.51%)	47(16.10%)	0.113
chronic bronchitis	69(24.82%)	86(29.45%)	0.214
tumor marker			
CEA(≥ 5 ng/ml)	4(1.44%)	1(0.34%)	0.206
NSE(≥ 16.3 ng/ml)	16(5.76%)	14(4.80%)	0.537
Cyfra21-1(≥ 3.3 ng/ml)	5(1.80%)	1(0.34%)	0.115
types of lung cancer	18(6.47%)	23(7.88%)	**0.038**
glandular cancer	2(0.72%)	20(6.85%)	**0.000**
squamous carcinoma	14(5.02%)	0(0.00%)	**0.000**
others	2(0.72%)	3(1.03%)	1.000

### Consistency between expert panel and IRCL to pulmonary nodules

**Fig.1:**
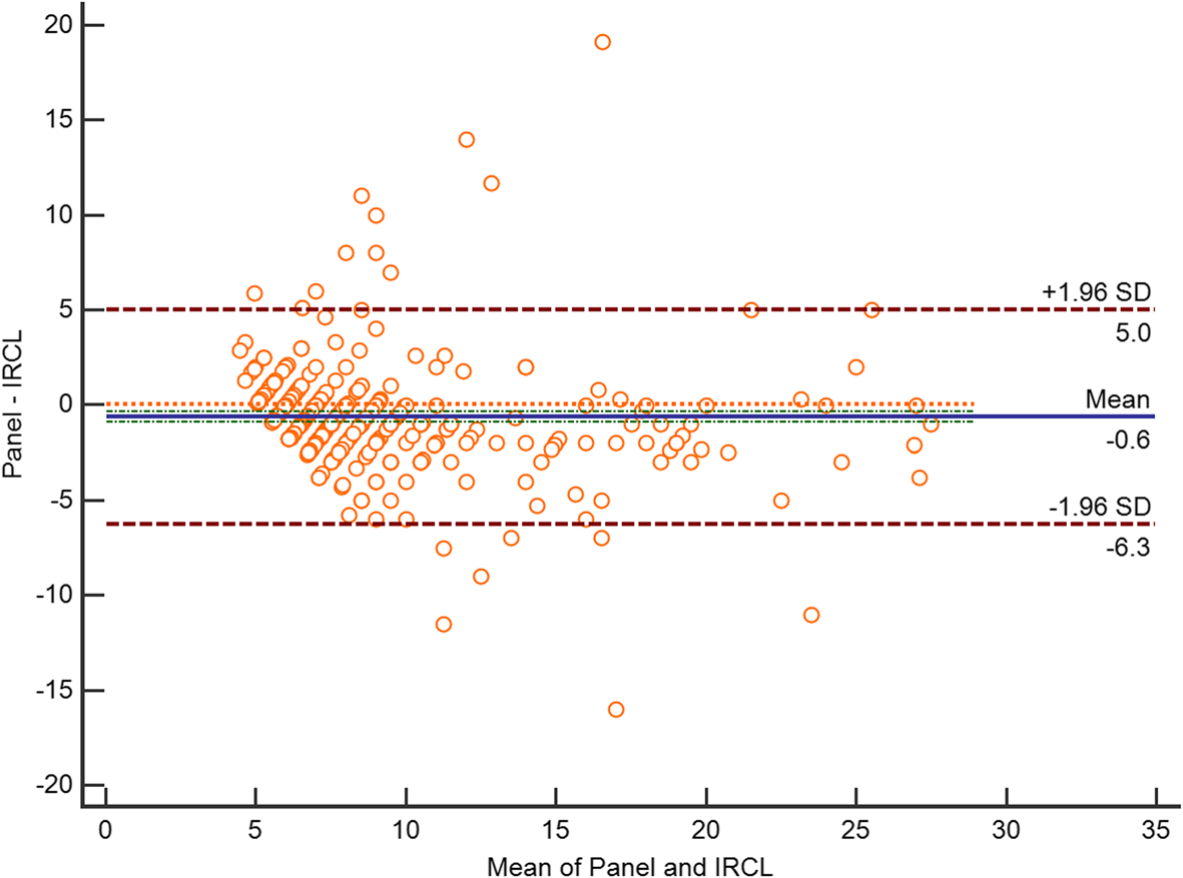
Consistency for the maximum diameter of pulmonary nodules measured by expert panel and the IRCL

Thin-section CT images of 570 subjects were analysed by the expert panel and IRCL, respectively. The majority of manually interpreted pulmonary nodules were located in the upper right lobe of the lung (18.25%, 104/570). Similarly, the IRCL interpreted the majority of pulmonary nodules in the upper right lobe of the lung (15.61%, 89/570), with strong agreement between the two on the localization of pulmonary nodules in the lobe (kappa = 0.883). Additionally, more pulmonary nodules were evaluated by the expert panel in the middle lobe of the right lung than by the IRCL (χ^2^ = 27.132, *p* = 0.000). The pulmonary nodules diameter (mean ± standard deviation(SD)) was 8.67 ± 4.41 mm by doctors, and the IRCL measured the diameter of 9.37 ± 4.90 mm. Moreover, the diameter of the IRCL measured was longer than the doctors (*p* = 0.002). There was a strong agreement between the panel and IRCL measurements of diameter (CCC = 0.809, *p* = 0.000), with a maximum difference of up to 5 mm between the two methods. (Fig. 1).

In a comparison of numbers, solid lung nodules were the most common type of pulmonary nodules in both doctor-read and IRCL readings (χ^2^ = 245.895, *p* = 0.000). The expert panel measured more solid and non-solid nodules compared to IRCL (χ^2^ = 18.227, *p* = 0.000; χ^2^ = 35.470, *p* = 0.000). Both methods demonstrated moderate concordance in the assessment of solid nodules (kappa = 0.503), while other attenuation features performed reasonably well. However, the panel and AI assessment performed poorly in interpreting morphological descriptions. Doctors identified more lobulation signs and pleural indentation signs than the software in morphologic characteristics (χ^2^ = 5.630, *p* = 0.018; χ^2^ = 131.797, *p* = 0.000). (Table 2).

**Table 2:** Consistency between expert panel and IRCL to pulmonary nodules

	Expert panel *n* = 570	IRCL *n* = 570	*p* value	kappa
**location**				0.883
upper lobe of right lung	104(18.25%)	89(15.61%)	0.236	0.851
middle lobe of right lung	61(10.70%)	55(9.65%)	**0.000**	0.867
inferior lobe of right lung	78(13.68%)	82(14.39%)	0.722	0.890
upper lobe of left lung	46(8.07%)	63(11.05%)	0.087	0.898
inferior lobe of left lung	80(29.63%)	81(14.21%)	0.932	0.935
**maximum diameter** (mm, mean ± SD)	8.67 ± 4.41	9.37 ± 4.90	**0.002**	
**attenuation characteristics**				0.360
solid nodules	308(54.04%)	236(41.40%)	**0.000**	0.503
subsolid nodules	89(15.61%)	86(15.09%)	0.805	0.242
nonsolid nodules	103(18.07%)	37(6.50%)	**0.000**	0.309
**morphologic characteristics**				
spiculation sign	98(17.18%)	81(14.21%)	0.166	0.102
lobulation sign	120(21.05%)	89(15.61%)	**0.018**	0.123
pleural indentation sign	135(23.68%)	7(1.23%)	**0.000**	0.004

### Diagnosis and prediction of lung cancer by expert panel and IRCL

**Fig.2:**
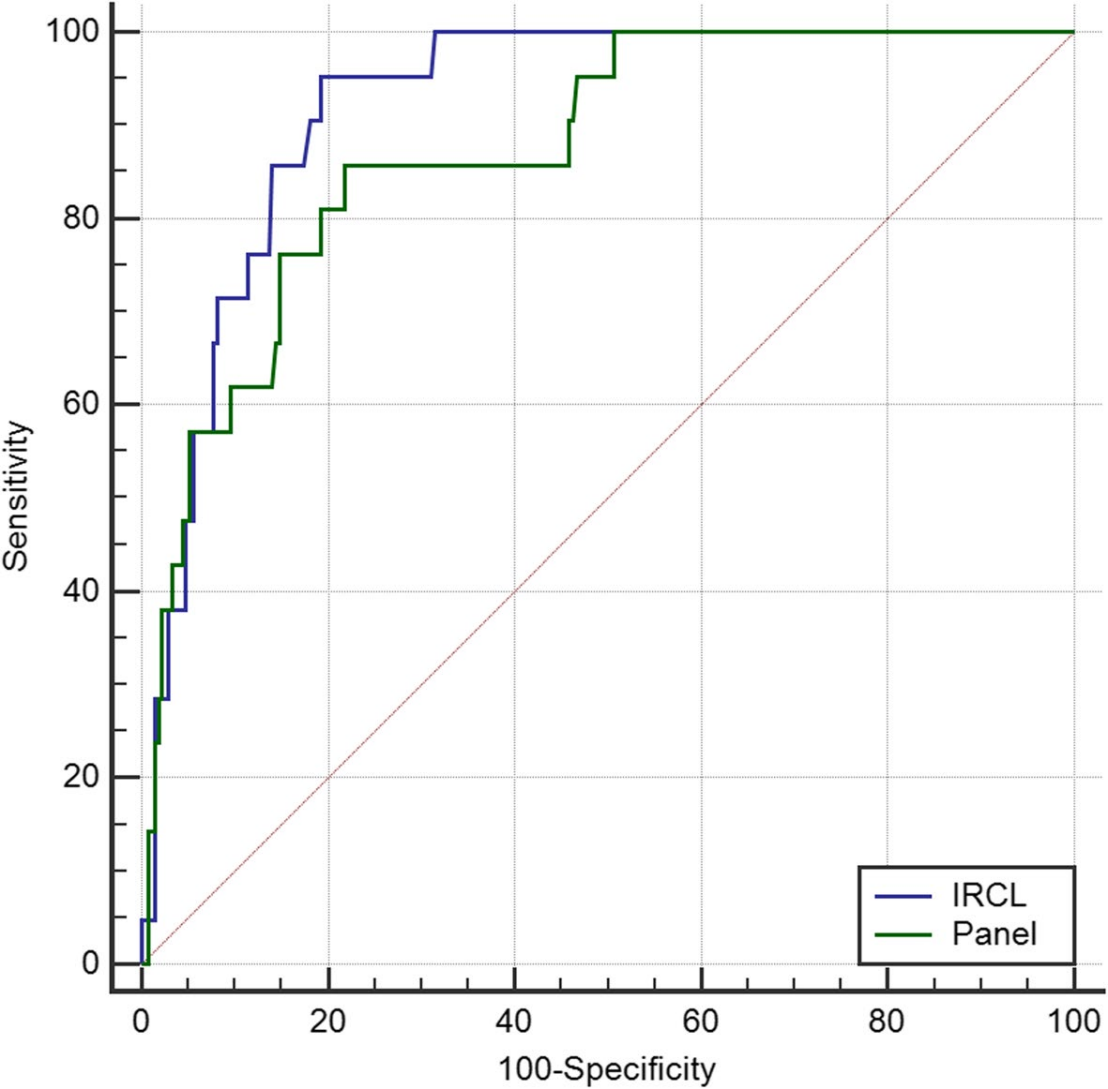
Comparison of ROC curve by expert panel and IRCL

When the data of Beijing adults aged 40–74 years, 21 out of 41 lung cancer patients underwent both expert panel and IRCL ROC analysis due to missing data. The AUC of the expert panel diagnosis for lung cancer was 0.873 (95% CI: 0.829–0.909), while the AUC of IRCL was 0.921 (95% CI: 0.884–0.949). The difference in the AUC for ROC between the two methods was 0.0486 (Fig. 2). However, the difference in the diagnostic value of the two methods was not statistically significant (*p* = 0.0950).

In this study, univariate and multivariable logistic regression were used to analyse whether the expert panel and IRCL pulmonary nodule characteristics (location, maximum diameter, attenuation features, and morphological characteristics) were significant factors in the interpretation of carcinomatous nodules based on the pathological findings of the screened patients (Table 3). The features of maximum diameter, solid nodules, subsolid nodules, spiculation sign, and lobulation sign were found to be statistically significant in the univariate logistic regression for both the expert panel and IRCL interpretation. In the multivariable logistic regression analysis for expert panel interpretation of nodal information, the maximum diameter (*p* = 0.000), solid nodules (*p* = 0.015), and subsolid nodules (*p* = 0.005) were identified as significant factors in determining carcinomatous nodules. Similarly, in the multifactorial logistic regression for IRCL, the maximum diameter (*p* = 0.016), solid nodules (*p* = 0.009), and subsolid nodules (*p* = 0.002) were statistically significant. The features of maximum diameter, solid nodules, and subsolid nodules were significant factors in the interpretation of cancerous nodules, as indicated by multifactorial regression analysis in both the expert panel and IRCL.

**Table 3:** Univariate and multivariable logistic regression were used to analyze for expert panel and IRCL lung nodule characteristics

Predictive factors	Expert panel				IRCL			
	Univariate logistic regression		Multivariable logistic regression		Univariate logistic regression		Multivariable logistic regression	
	OR(95%CI)	*p* value	OR(95%CI)	*p* value	OR(95%CI)	*p* value	OR(95%CI)	*p* value
upper lobe of right lung	-	0.973	-	-	-	0.659	-	-
middle lobe of right lung	0.000(0.000–0.000)	0.997	-	-	0.000(0.000–0.000)	0.977	-	-
inferior lobe of right lung	1.041(0.370–2.928)	0.940	-	-	1.093(0.366–3.263)	0.873	-	-
upper lobe of left lung	1.005(0.293–3.448)	0.993	-	-	1.952(0.686–5.556)	0.210	-	-
inferior lobe of left lung	0.704(0.226–2.188)	0.544	-	-	0.937(0.301–2.914)	0.911	-	-
maximum diameter	36.259(8.658–151.849)	**0.000**	23.098(5.399–98.814)	**0.000**	28.326 (3.811–210.508)	**0.001**	12.713(1.608–100.507)	**0.016**
solid nodules	-	**0.000**	-	**0.015**	-	**0.001**	-	**0.009**
subsolid nodules	5.926(2.708–12.967)	**0.000**	3.757(1.494–9.446)	**0.005**	4.599(2.094–10.100)	**0.000**	3.886(1.628–9.277)	**0.002**
nonsolid nodules	1.845(0.810–4.200)	0.145	1.381(0.551–3.461)	0.492	0.000(0.000–0.000)	0.998	0.000(0.000–0.000)	0.998
spiculation sign	3.059(1.475–6.345)	**0.003**	1.062(0.369–3.061)	0.911	7.307(3.747–14.250)	**0.000**	254009962(0.000–0.000)	0.999
lobulation sign	3.961(1.955–8.026)	**0.000**	2.340(0.862–6.352)	0.095	6.349(3.273–12.316)	**0.000**	0.000(0.000–0.000)	0.999
pleural indentation sign	0.953(0.421–2.158)	0.907	-	**-**	18.955(4.090–87.845)	**0.000**	3.174(0.585–17.207)	0.181

## Discussion

This study was a retrospective analysis of lung cancer screening among residents aged 40–74 years in Beijing from 2011 to 2013. It involved 570 subjects who met the inclusion–exclusion criteria and aimed to compare the differences between the expert panel and IRCL analyses of pulmonary nodules larger than 5 mm. The expert panel assessment showed strong agreement with IRCL measures in pulmonary nodules localization and diameter (kappa = 0.878; CCC = 0.809, *p* = 0.000). It also demonstrated moderate agreement in comparisons of attenuation characteristics (kappa = 0.503 vs. kappa = 0.242 vs. kappa = 0.309). For patients diagnosed with lung cancer based on pathology, the AUC of IRCL was higher than that of the expert panel (0.921 vs. 0.873), although the difference in diagnostic value between the two methods was not statistically significant (*p* = 0.095).

The potential usefulness of AI in disease diagnosis is widely recognized, but its agreement with doctors has not yet been substantially demonstrated in pulmonary nodule screening. In this study, the kappa value of 0.878 between the IRCL and doctors' localization of ≥ 5 mm pulmonary nodules in the screening population indicated strong agreement. However, a CAD-based pulmonary nodule software for 100 patients with pulmonary nodules exhibited only moderately strong concordance(kappa = 0.44) compared to radiologists' interpretation for pulmonary nodules localization [10]. Nodule size is a strong predictor of lung cancer [11]. In pulmonary nodule diameters, this study also demonstrated good agreement between IRCL and the expert panel assessments, indicating excellent spatial overlap with manual segmentation of pulmonary nodules. The initial application of AI in medical imaging focused on automating the detection of pulmonary nodules. Some studies have indicated that its performance in this area has even surpassed that of radiologists [12]. In this study, the detection rate of subsolid nodules by IRCL was comparable to that of the expert panel. However, IRCL's detection rate was lower than that of doctors for solid and non-solid nodules. This difference was speculated to be due to the exclusion criteria of the subjects and the localization of pulmonary nodules that were not within the lung segments. Upon analysing morphological characteristics, it was found that the consistency of the spiculation sign, lobulation sign, and pleural indentation sign was all low. A study by Smith et al. [10] similarly demonstrated the spiculation sign for pulmonary nodules with a lower consistency (kappa = 0.14).

An additional application of AI involves classifying pulmonary nodules as benign or malignant. In an analysis of the accuracy of pulmonary nodules diagnosed as lung cancer based on the pathological gold standard, the AUC of IRCL for detecting pulmonary nodules as malignant was not significantly different from the expert panel comparison (0.921 vs. 0.873, *p* = 0.095). This indicates that the IRCL was not inferior to the doctors' judgment of the benign or malignancy of the pulmonary nodules. Espinoza et al. [13]. concluded that the algorithms can achieve accuracy comparable to an experienced radiologists. The CNNs demonstrated strong agreement with radiologists' diagnoses of pulmonary nodules in a study on the accuracy of pulmonary nodule detection. (kappa = 0.846) [14].

In this study, the analysis of factors in the interpretation of cancerous nodules by an expert panel and IRCL characteristics for pulmonary nodules revealed that the maximum diameter was identified as a statistically significant variable, as well as an important interpretation factor of cancerous nodules in multivariable logistic regression. Farjah et al. [15] reported that the diameter of the pulmonary nodule was a factor associated with the diagnosis of lung cancer in lung cancer screening, this study yielded similar results. Moreover, the predictive role of pulmonary nodule size was the same as the results of prospective studies [16]. Schreuder et al. [9] reported that the larger nodule size is currently the best CT predictor of malignancy. The subsolid nodules were an important factor in the development of lung cancer [17], the subsolid nodules were all statistically significant factors in the expert panel and IRCL in the univariate analysis of the interpretation of carcinomatous nodules, which is consistent with the results of this study.

In this screening study for pulmonary nodules, more women than men had lung cancer (χ^2^ = 4.321, *p* = 0.000), and more women than men had adenocarcinoma (χ^2^ = 14.421, *p* = 0.000), and Smeltzer et al. found the similar result in lung cancer screening study [18].

The limitations of our study also merit acknowledgment. Firstly, this study aimed to screen for pulmonary nodules in individuals aged 40 years or older in Beijing. Only a baseline low-dose chest CT scan was conducted at the time of inclusion in the study, with no follow-up performed, resulting in no additional information available for analysis and discussion. In clinical practice, pulmonary nodules were initially localized to the lung segment before further description. However, this study localized the pulmonary nodules at the lobe level, which has a limiting role in the analysis of the precise localization. Furthermore, multiple pulmonary nodules were not analysed in this study. Finally, the conclusions of this study could be verified using AI software on patients screened for pulmonary nodules in recent years, which could be investigated in the future.

In conclusion, AI software is consistent with doctors' evaluations and diagnoses of pulmonary nodules. AI software can assist clinicians in detecting pulmonary nodules of lung cancer screening programs.

## Data Availability

The datasets used and/or analysed during the current study available from the corresponding author on reasonable request.
